# Mechanical Characterization of Nanocomposite Joints Based on Biomedical Grade Polyethylene under Cyclical Loads

**DOI:** 10.3390/polym12112681

**Published:** 2020-11-13

**Authors:** Annamaria Visco, Cristina Scolaro, Antonino Quattrocchi, Roberto Montanini

**Affiliations:** 1 Department of Engineering, University of Messina, C.da di Dio, 98166 Messina, Italy; cristina.scolaro@unime.it (C.S.); antonino.quattrocchi@unime.it (A.Q.); roberto.montanini@unime.it (R.M.); 2 Institute for Polymers, Composites and Biomaterials-CNR IPCB, Via Paolo Gaifami 18, 95126 Catania, Italy

**Keywords:** nanocomposite, UHMWPE, titanium dioxide, laser welding, hardness, tensile test, fatigue limit

## Abstract

Polymeric joints, made of biomedical polyethylene (UHMWPE) nanocomposite sheets, were welded with a diode laser. Since polyethylene does not absorb laser light, nanocomposites were prepared containing different percentages by weight of titanium dioxide as it is a laser absorbent. The joints were first analyzed with static mechanical tests to establish the best percentage weight content of filler that had the best mechanical response. Then, the nanocomposites containing 1 wt% titanium dioxide were selected (white color) to be subjected to fatigue tests. The experimental results were also compared with those obtained on UMMWPE with a different laser light absorbent nano filler (carbon, with greater laser absorbing power, gray in color), already studied by our research team. The results showed that the two types of joints had an appreciable resistance to fatigue, depending on the various loads imposed. Therefore, they can be chosen in different applications of UHMWPE, depending on the stresses imposed during their use.

## 1. Introduction

For almost half a century, polymer and composite joints have been largely studied by several fields of industry. Some applications need welding methods to seal two or more different parts of plastic materials. Good quality production depends on the welding technique and on the weldability of the polymeric materials. Consequently, the common purpose is to identify the chemical–physical changes that occur during the welding processes to be able to guarantee the best joint performance [1,2]. Polymers are transparent to the irradiation sources used to seal two or more plastic components [3]. A recent practice is to create polymer-based nanocomposites by adding nanoparticles of different materials that are capable of absorbing the light from the irradiation source (e.g., clay, carbon, silver, etc.) to a polymeric matrix (e.g., polypropylene, polyethylene, polyamide, etc.). These contaminations, usually called a “filler”, are intended to enhance the weldability of the base material and consequently improve the joint resistance [4,5,6].

Joints, obtained by these advanced materials, require innovative welding techniques such as laser welding. It is a non-contact, non-contaminating, precise, and flexible melt process that can be easily controlled and automated to join thermoplastics [7]. The joining process is based on the melting caused by the heating associated with the absorption of radiation by the polymeric material. Three different types of laser beam can be used: diode, CO_2_, or Nd:YAG [8,9,10,11]. Specifically, the two or three sheets of materials are often subjected to pressure to improve the contact zone. However, the main obstacle of this method on thermoplastics is the high intensity of the laser beams, which easily burn the polymers, even at relatively low power levels [12,13].

The biomedical industry represents an interesting field of applications of laser welded nanocomposite joints with a polymeric matrix. For example, Amanat et al. [14] reported how these types of materials are widely employed to create hermetic enclosures for medical devices. In fact, although thermoplastics allow for a reduction in the contamination of prosthesis, they are permeable to gases including water vapor. Authors have highlighted that their nanocomposites did not have this limit, and also showed good mechanical strength of the joint.

To the best of our knowledge, there is limited literature on evaluating the mechanical behavior of laser welded polymer and composite joints. Furthermore, almost all articles focused on the evaluation of the static mechanical properties and very few on the dynamic ones. Recently, Pereira [15] assessed the tensile strength of the Nd:YAG laser welded polyamide joints in three different configurations: 45° scarf, single, and double lap. The results showed that the welded area was reduced when compared to the diameter of the laser beam due to the transmission process, and that with the same pass, the scarf joint was more efficient than the lap ones. Several researchers have underlined that the design of the welding process for a polymeric material represents a necessary consideration as welding parameters such as laser power, welding speed, laser beam diameter, laser beam width, and joint overlapping pressure determine the achievable mechanical performance [16,17]. However, for composite joints, it is necessary to consider that the technique is not perfectly controllable due to the random dispersion of the filler in the composite matrix. The previous consideration was estimated by Votrubec et al. [18] on a limited sampling of glass fiber-polyethylene joints. Hence, the filler dispersion degree within the polymeric matrix is a crucial point to achieve the welding of the perfect material and a consequent appreciable mechanical response.

One of the first mechanical studies on nanocomposite polymeric joints, in particular based on polypropylene and polypropylene with 0.5 wt% of carbon black, was presented by Spancken et al. [19]. They defined specific equipment to study hermetically sealed specimens as able to define the influences of the different weld geometries and the static and cyclic load, in terms of pressure, on the weld line strength.

In our previous paper [20], we manufactured and analyzed joints of two sheets of pure ultra-high molecular weight polyethylene (UHMWPE) and doped with a nano filler at different concentrations (carbon, titanium, and silver nanoparticles), welded by a diode laser. Single lap and double lap geometries were investigated by identifying the best performances by means of static (shear stress) and dynamic analysis. Recently, another investigation of ours [21] focused on the dynamic behavior of joints consisting of two biomedical grade UHMWPE sheets doped with carbon nanoparticles. The fatigue limit was estimated, and the fracture of the joints was analyzed through Scanning Electron Microscope (SEM) characterization.

In this work, considering the lack of information on the dynamic mechanical response of polymeric joints, we analyzed the fatigue behavior of the ultra-high molecular weight polyethylene (UHMWPE) mixed with the TiO_2_ nanofiller and welded by a diode laser. The TiO_2_ nanofiller was chosen among the possible laser absorbing fillers in order to retain the white color of polyethylene and, hence, for aesthetic reasons. Static tests (shore-D hardness and lap shear test) have been preliminarily performed to define the correct filler amount in the nanocomposite to obtain the best mechanical response and the consequent method for dynamic tests. A correlation between the filler dispersion degree and the mechanical static response has been evidenced. Finally, fatigue behavior of the TiO_2_/UHMWPE nanocomposites studied in this paper were compared with the same fatigue results of a carbon nanofiller or the CNF/UHMWPE nanocomposite analyzed in our previous paper [21], with the aim to establish the correlation between the material’s properties and the mechanical response.

## 2. Materials and Methods

**Figure 1:**
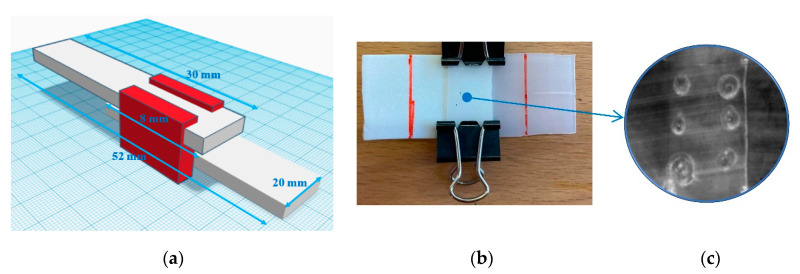
Scheme and geometry of a typical junction (**a**) made by two overlapped polymer sheets blocked by the clamps (image not in scale) (**b**); welded area in the single lap joint (SLJ) obtained by six laser spots observed by an infrared camera (MWIR 3.6–5.1 μm) as reported in [23] (image not in scale (**c**).

White titanium dioxide powder (TiO_2_, purchased from Sigma-Aldrich, St. Louis, MO, USA) had an average particle size of 50–100 nm. The TiO_2_ was mixed in different quantities within the range of 0.5–6.0 wt% (as listed in Table 1) with a polymeric powder of the same milky-white color typical of this polymer (UHMWPE, Ticona-GUR 1020, Sulzbach, Germany). The powder size of UHMWPE was 150 nm, ρ = 0.930 g/cm^3^, Mw ≈ 3 × 10^6^ g/mol). Pure ethanol (purchased from Honeywell Fluka, Morristown, NJ, USA) was used as a solvent to disperse the filler inside the matrix with the help of an ultrasonic bath for 2 h at an average room temperature of 25 °C. The residual ethanol was then removed by magnetic stirring with a hot plate. The resulting powder, also white in color, was compressed at T = 200 °C for 20 min and P = 200 atm to obtain a sheet whose rectangular geometry was 30 mm × 20 mm, and 0.5 mm thick. Two sheets were partially overlapped for a length of 8 mm and interlocked to obtain a single lap joint (SLJ), as schematized in Figure 1a. The SLJs were sealed by a diode laser (purchased by Lambda Scientific- mod. D5 Doctor Smile 970 nm, Brendola, Italy) with a fiber diameter of 300 μm, a maximum laser pulse energy of 100 mJ, and irradiation times of the order of 120–150 s. More details about the laser welding technique have been discussed in a previous paper of ours [22].

**Figure 2:**
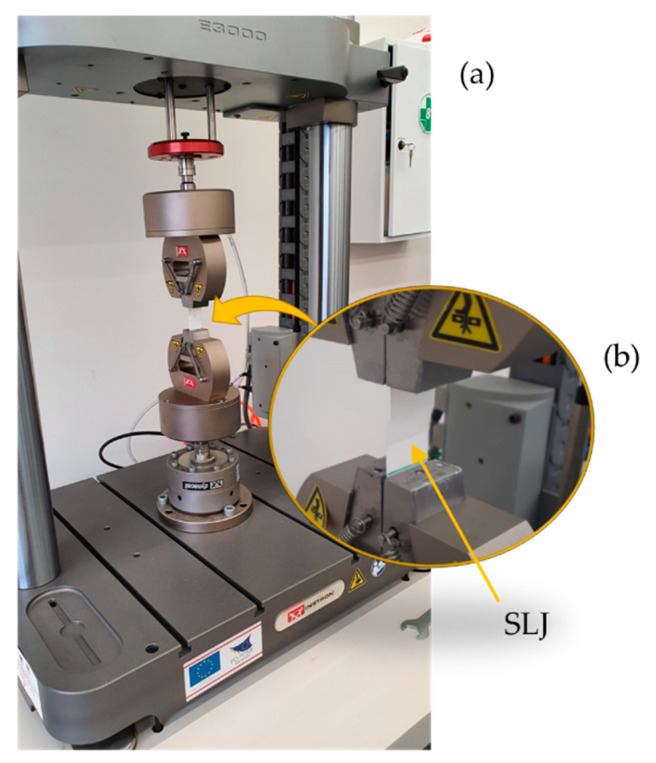
Experimental setup for fatigue tests (**a**). In the frame, the joint locked by the pneumatic grips is shown (**b**).

Two clamps blocked the overlapped sheets, exerting a pressure of 30 N in the joint area in order to favor the maximum sealing power between the two polymer sheets in the overlapped area (Figure 2b).

An image of the welded area is shown in Figure 1c where a total number of six welding points were made in the whole welding area to ensure a good sealing action between the two overlapped sheets.

Preliminary static tests were the surface hardness (as the ASTM D 2240 standard), evaluated by PCE-HT 210 (resolution: 0.1° of hardness, accuracy: ±1°, scale range: 0–100°), and the lap shear test (as ASTM D638 standard) performed by a Lloyd LR10K (Lloyd Instruments, Bognor Regis, UK) with a load cell of 500 N and the traverse rate of 5 mm/min. The mechanical parameters were generally the average of sixteen samples. Both hardness and lap shear tests were performed at the room temperature of 25 °C. Typical load/displacement curves of shear stressed joints were obtained and each mechanical parameter was the result of an average of at least six specimens.

The mean differences and the standard deviations of the Shore D analysis and of the tensile test of all the samples with different amounts of TiO_2_ were evaluated using the Prism 8.0.2 program; GraphPad Software, Inc, La Jolla, CA, USA. The data were first verified with the Augustine & Pearson test for the normality of distribution and the Levene test for the homogeneity of variances. Since the data were found to be normally distributed and homogeneous, they were subsequently statistically analyzed using two-way analysis of variance (ANOVA) and Bonferroni post-hoc test for multiple comparisons at a significance level set at *p* < 0.05.

For dynamic tests, the experimental setup (Figure 2) consisted of an electromechanical testing machine (Electropuls E3000, Instron, Norwood, MA, USA) equipped with two pneumatic grips and a calibrated load cell of ±5 kN max. Fatigue tests were performed on 24 joints, according to the ASTM D7791 standard. A sinusoidal uniaxial stress of tension and compression in load control was applied to the sample, considering a stress ratio (R) of 0.7 and a frequency of 1 Hz at a room temperature of 25 ± 1 °C and humidity of 50 ± 10% RH. For each load level, three specimens were tested and the results were acquired at a sampling frequency of 100 Hz, using a specific cycle reduction mode: from the 0 to 1000 cycles, all were captured; between 1000 and 100,000 cycles, one in every four; and finally after 100,000 cycles of one in every 40, moreover, a specific software protocol was considered to acquire the last 100 cycles before the failure cycle.

## 3. Results and Discussion

**Figure 3:**
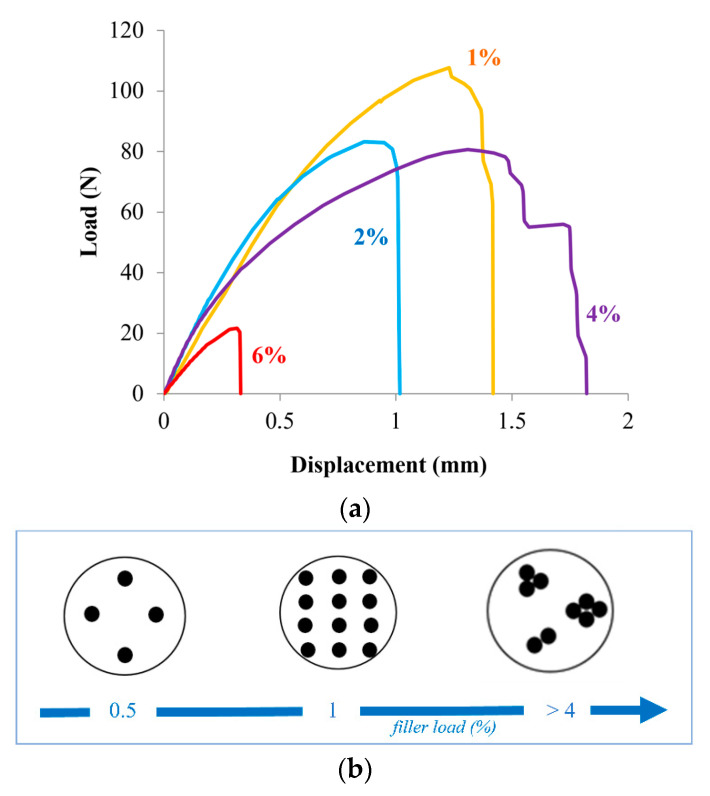
Load-displacement representative curves of the joints with different TiO_2_ amounts (**a**). Possible filler distribution within the polymeric matrix at different filler amounts (**b**).

Figure 3a shows the load/displacement trend of the joints with 1, 2, 4, and 6% of TiO_2_. As can be seen, the curves showed different trends depending on the percentage of filler. The main parameters are listed in Table 1. In particular, the highest breaking load was obtained in the sample with 1 wt% (108 N, *p* < 0.0001), while the sample at 6% showed the worst result (28 N, *p* < 0.0001). The sample with the filler load of less than one percent (i.e., 0.5 wt%) was not weldable and therefore could not be tested. This obviously means that the percentage of fillers lower than 1% are insufficient to be able to distribute themselves evenly within the polymeric matrix (which, as already said, is absolutely transparent to laser light) and therefore cannot absorb the energy needed to create an effective weld.

Similarly, high filler loads can be equally counterproductive because they create aggregation or clusters of nanoparticles due to their high quantity. Therefore, the filler remains poorly distributed, or not homogeneously distributed, within the polymer matrix. To give an idea of how the nanoparticles are distributed within the polymer matrix as a function of the percentage content of TiO_2_ filler, a graphic representation was made in Figure 3b. Filler amounts of 0.5 wt% or, more generally, lower than 1 wt%, represents a poor filler presence so that the matrix behavior is not really changed. Instead, a filler quantity higher than 4 wt% is too high and gives rise to the cluster formation of particles that randomly agglomerate within the polymer. The filler amount of about 1 wt% was the best condition where the particles were homogeneously distributed within the polymer matrix. In this way, the nanocomposite has a reinforcing action by the filler with a resulting overall improvement in the physical and mechanical features.

Therefore, the absorption of laser light cannot take place effectively in the composite with a filler quantity ≥4 wt%, and even in this case, it was not possible to make good joints. The best compromise occurred at average filler loads such as that of 1. In this sample, we could observe the best breaking load, an appreciable deformability, and the highest surface hardness value, according to the result observed with the tensile test and according to the inferential analysis that revealed statistically significant differences (*p* < 0.0001). From Table 1, we observed, in fact, that the Shore D hardness of pure UHMWPE (71.85 HD, *p* < 0.0001) increased with the increase in the filler content, as expected, with up to 1% reaching a maximum of 82.38 HD (*p* < 0.0001). As the percentage of filler increased further, it decreased to 74.77 HD (*p* < 0.0001) in the sample with 6% TiO_2_.

On the basis of the static mechanical results discussed above, it was therefore decided to carry out the fatigue test on SLJ, made with nanocomposites at 1 wt% of TiO_2_ and specifically codified with the abbreviation TiO_2_/UHMWPE.

**Figure 4:**
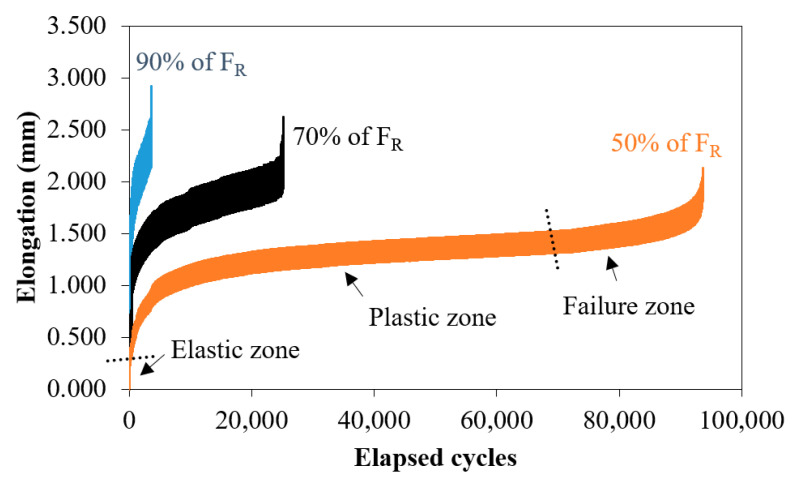
Typical elongations for the 1 wt% TiO2/UHMWPE samples subjected to 90%, 70%, and 50% of F_R_.

Figure 4 shows the typical elongations that occurred during the fatigue test as a function of the elapsed cycles for the nanocomposite TiO_2_/UHMWPE. Although the static breaking load of these joints had a mean value of 83 N, we decided to consider a more conservative condition, that is F_R_ = 78 N, to evaluate the fatigue behavior. Three different fractions of F_R_, 90%, 70%, and 50% (which correspond to 70.2 N, 54.6 N and 39 N, respectively) are reported.

All the acquired curves underline the three typical regions of fatigue: elastic, plastic, and failure zone [20]. With the load decreasing (up to 50%), the plastic zone presented an evident increase, compared to 90%, and the failure behavior occurred after a greater number of cycles (as indicated in the graph). These trends could be explained by the typical viscoelastic properties of polyethylene. Failure cycle values are listed in Table 2.

**Figure 5:**
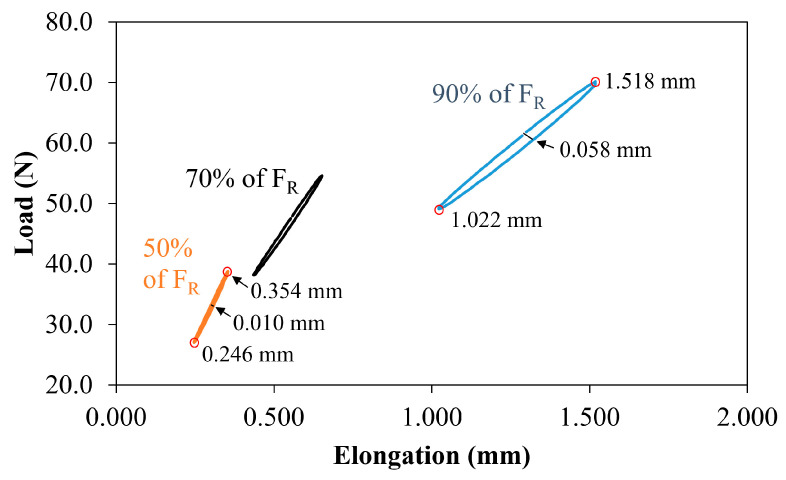
Typical hysteric cycles at the 100th cycles for the TiO_2_/UHMWPE samples (1 wt% of TiO_2_) subjected to 90%, 70%, and 50% of F_R_.

Figure 5 displays the 100th hysteric cycle for the TiO_2_/UHMWPE samples subjected to 90%, 70%, and 50% of F_R_. The area enclosed by the hysteric cycle represents the energy per unit of volume adsorbed by the sample during the load application and, consequently, the work produced by the action of the forces. Specifically, the cycle in the figure can be sufficiently considered as the starting point of the plastic deformation region.

The hysteric cycle area decreased with the reduction of the applied load, following a trend that would seem non-linear. In particular, the maximum and minimum values of the fatigue elongation and the amplitude of the hysteric cycle (the latter is calculated when the applied load is half the load amplitude) showed a reduction of about five times. In fact, the amplitude cycle decreased from 0.058 mm to 0.010 mm, as indicated in the graph.

**Figure 6:**
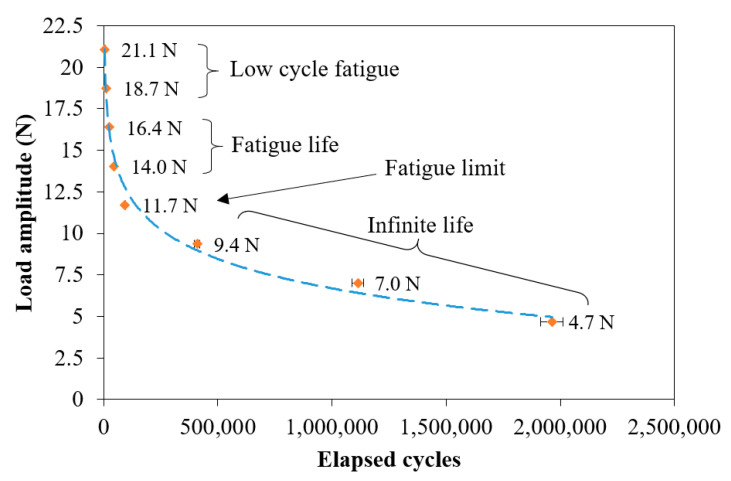
Whöler curve of the TiO_2_/UHMWPE joints with the different fatigue zones.

Figure 6 illustrates the Whöler curve for the TiO_2_/UHMWPE samples, considering eight levels of load amplitude (from 90% to 20% of F_R_ with 10% steps, Table 2). Each point was evaluated on three samples and represents the mean value of the elapsed cycles with the respective standard deviation. Table 2 lists the dynamic parameters of the fatigue test of the TiO_2_/UHMWPE joints with the aim to report the data for the ASTM D7791 standard specifications (% of F_R_, maximum load, minimum load, and load amplitude) and the failure cycles with their standard deviation.

The different fatigue zones are specified inside Figure 6. The low cycle fatigue zone was barely visible between the amplitudes of 21.1 N and 18.7 N, up to about 10,000 cycles, while the finite life fatigue zone was more evident and extended up to the amplitude of 14 N, at about 45,000 cycles. The fatigue limit can be acceptably identified at the amplitude of 11 N at about 95,000 cycles. Then, the infinite life begins.

The reported fatigue results are consistent with those of than in our previously published papers [20,21]. In these previous works, joints made of the same UHMWPE filled with carbon nanoparticles and welded by the same diode laser were statically and dynamically tested. In particular, the best mechanical performance was obtained for the DLJ with a 0.016 wt% of carbon nanofiller (here generally codified as CNF/UHMWPE), and its fatigue trend was achieved using a similar testing method to that applied in the paper.

The two type of joints displayed a different elongation behavior, thus, as we can see from the experimental results, CNF are more effective than TiO_2_. In fact, CNF produces an effective welded joint with a lower filler content (0.016 wt%) with respect to TiO_2_ (1 wt%). This is possible because the laser-absorbing power of CNF (coefficient absorption light α = 82.33 cm^−1^ at IR wavelengths) is greater than TiO_2_ α = 79.16 cm^−1^ (always at IR wavelengths) [22]. Another cause can be identified in the filler–matrix interactions and the consequent filler dispersion, especially considering the different chemical nature of the components. In particular, the apolar character of CNF and the polar one of TiO_2_ could be differently related to apolar polyethylene, resulting in a favorable chemical interaction and better dispersion of the apolar CNF filler compared to the polar TiO_2_ one.

**Figure 7:**
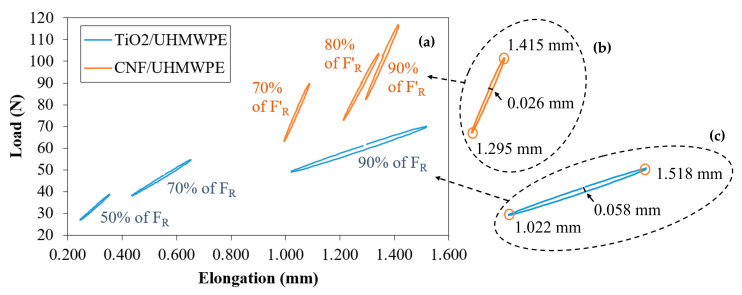
Comparison of the typical hysteric cycles of SLJ filled with CNF/UHMWPE (50 cycles) and TiO_2_/UHMWPE samples (100 cycles) (**a**), with a magnification of the cycle at 90% of F’_R_ for CNF/UHMWPE samples (**b**) and of the cycle at 90% of F_R_ for TiO_2_/UHMWPE (**c**).

The static test highlighted that the CNF/UHMWPE features were higher than that of TiO_2_/UHMWPE (see Table 3). The typical hysteric cycle (Figure 7) of 90% of F_R_ for TiO_2_/UHMWPE has an elongation range to include those of 70%, 80%, and 90% of F′_R_ for CNF/UHMWPE (F′_R_ is the reduced fatigue load for CNF/UHMWPE, which corresponds to 117.0 N). Furthermore, the amplitude of the same hysteric cycle of 90% of F_R_ for TiO_2_/UHMWPE (0.026 mm) was more than double that of 90% of F′_R_ for CNF/UHMWPE (0.058 mm), which were well visible in the external frames of Figure 7a,b. However, it should be considered that these trends were observed for different cycles: 50th for CNF/UHMWPE and 100th for TiO_2_/UHMWPE. This observation did not make the comparison any less effective since the total cycles in the elastic region of CNF/UHMWPE were almost half that of TiO_2_/UHMWPE. All these observations underline that TiO_2_/UHMWPE is more ductile than CNF/UHMWPE, even considering that the first was subjected to more reduced loads than the second one.

**Figure 8:**
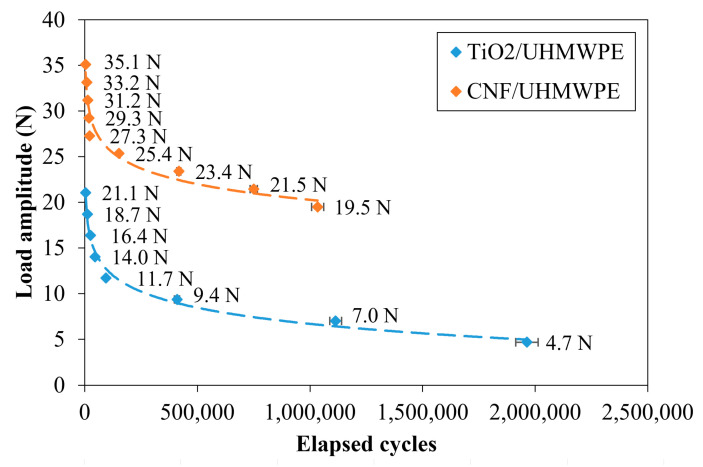
Comparison of the Whöler curve of the two CNF/UHMWPE and TiO_2_/UHMWPE SLJs.

Finally, the TiO_2_/UHMWPE fatigue limit was about five times that of CNF/UHMWPE. In particular, the fatigue limit of TiO_2_/UHMWPE (Figure 8) was about five times that of CNF/UHMWPE (95,000 vs. 22,000 cycles, respectively). However, it must be considered that the loads to which the joints were subjected were not the same (~11 N and ~27 N, respectively), as listed in Table 3. Figure 7 compares the Wöhler curves of the TiO_2_/UHMWPE and CNF/UHMWPE samples [17].

Considering that the two types of joints were manufactured with the same overlap (double lap), and above all had the same dimensions, at about 21 N, the CNF/UHMWPE was abundant in its condition of infinite fatigue life, while TiO_2_/UHMWPE had low cycles of fatigue zone. It can be deduced that CNF/UHMWPE exhibited better dynamic performances than TiO_2_/UHMWPE.

The disadvantage of the CNF/UHMWPE joint is a change in color, from white to heavy gray, while TiO_2_/UHMWPE retained the typical milk-white color of the UHMWPE. In conclusion, high performance fatigue applications need the choice of carbon filler with little color changes, while low performance fatigue use does not require any color change of the material because of the employment of the TiO_2_ filler.

## 4. Conclusions

In this work, the mechanical behavior of nanocomposite joints welded by a diode laser, were investigated. Nanocomposites were made by biomedical grade polyethylene (UHMWPE) and laser absorbent TiO_2_ filler. This plastic is of great industrial interest for properties such as biocompatibility, chemical inertia, and high ductility. Notoriously, it resists corrosion and radiation, and is an electrical insulator. It has excellent wear resistance characteristics, thanks to its good abrasion and impact resistance. Thus, UHMWPE has wide applications in the electronics, pharmaceutical, and medical fields where welded counterparts are produced in several components.

Static properties were performed on nanocomposites with different filler amounts and they highlighted that the optimal value was 1 wt% of nanoparticles in order to achieve good weldability.

In this case, the lap shear mechanical tests showed results in agreement with the surface hardness results. Consequently, the mechanical fatigue test was performed on the sample with 1 wt% of TiO_2_ and indicated a fatigue limit of about 95,000 cycles, obtained with a load of about 11 N. This joint (TiO_2_/UHMWPE) was compared in terms of static and dynamic performance with a previously studied joint containing 0.016 wt% CNF (carbon filler). Considering a chromatic point of view, the CNF/UHMWPE joint was light gray, while TiO_2_/UHMWPE showed the typical white color of pure UHMWPE. However, mechanically, the CNF/UHMWPE joint had both a higher static and dynamic strength with respect to the TiO_2_/UHMWPE with a mean max static failure load of 118 N versus 83 N and a fatigue limit of about 22,000 cycles versus 95,000 cycles. The peculiar mechanical behavior of the investigated materials suggests that they could be used for different application types, depending on the load they will have to bear.

## Figures and Tables

**Table 1:** Static hardness and tensile test of the nanocomposite joints at different percentages of filler. For a filler amount of 0.5 wt%, the weld process was not obtained.

TiO_2_ Amount (wt%)	Shore D Hardness (HD)	Failure Load (N)	Displacement (mm)	Work at Break (J)
0	71.85 ± 0.4	-	-	-
0.5	77.23 ± 0.35	-	-	-
1.0	82.38 ± 0.31	108 ± 5.3	1.42 ± 0.09	0.34 ± 0.01
2.0	80.87 ± 0.67	83 ± 6.9	1.02 ± 0.04	0.14 ± 0.02
4.0	79.77 ± 0.65	81 ± 2.3	1.82 ± 0.08	0.20 ± 0.03
6.0	74.77 ± 0.53	28 ± 1.6	0.33 ± 0.01	0.0035 ± 0.0001

**Table 2:** Dynamic parameters of the fatigue tests of the TiO_2_/UHMWPE joints (1 wt% of TiO_2_).

% of F_R_(N)	Max Load(N)	Min Load (N)	Load Amplitude(N)	Failure Cycle	St. Dev. Failure Cycles
90	70.2	49.1	21.1	3562	37
80	62.4	43.7	18.7	11,444	232
70	54.6	38.2	16.4	25,172	394
60	46.8	32.8	14	45,396	903
50	39	27.3	11.7	93,626	2,674
40	31.2	21.8	9.4	40,9528	11,322
30	23.4	16.4	7	1,113,827	25,764
20	15.6	10.9	4.7	1,963,485	49,452

**Table 3:** Comparison of the static and dynamic performance of the TiO_2_/UHMWPE and CNF/UHMWPE samples. The values of the fatigue properties are indicative. CNF/UHMWPE dynamic characterization is reported in [21].

Filler Code	Amount wt%	Shore D Hardness (HD)	Static Failure Load (N)	Fatigue Limit (cycle)	Fatigue Limit Max Load (N)
TiO_2_/UHMWPE	1	80.87	83 ± 6.9	~95,000	~11
CNF/UHMWPE	0.016	73.38	169 ± 4	~22,000	~27
